# Development of New Microsatellite Markers for *Salvia officinalis* L. and Its Potential Use in Conservation-Genetic Studies of Narrow Endemic *Salvia brachyodon* Vandas

**DOI:** 10.3390/ijms130912082

**Published:** 2012-09-24

**Authors:** Ivan Radosavljević, Zlatko Satovic, Jernej Jakse, Branka Javornik, Danijela Greguraš, Marija Jug-Dujaković, Zlatko Liber

**Affiliations:** 1 Department of Botany, Division of Biology, Faculty of Science, University of Zagreb, Marulićev trg 9A, 10000 Zagreb, Croatia; E-Mails: ivanrad@botanic.hr (I.R.); danijela_greguras@hotmail.com (D.G.); 2 Department of Seed Science and Technology, Faculty of Agriculture, University of Zagreb, Svetošimunska 25, 10000 Zagreb, Croatia; E-Mail: zsatovic@agr.hr; 3 Centre for Plant Biotechnology and Breeding, Agronomy Department, Biotechnical Faculty, University of Ljubljana, Jamnikarjeva 101, 1000 Ljubljana, Slovenia; E-Mails: jernej.jakse@bf.uni-lj.si (J.J.); branka.javornik@bf.uni-lj.si (B.J.); 4 Institute for Adriatic Crops and Karst Reclamation, Put Duilova 11, 21000 Split, Croatia; E-Mail: masa@krs.hr

**Keywords:** common sage, conservation, cross-amplification, Dalmatian sage, genetic bottleneck, microsatellites, Lamiaceae, population genetics, short-tooth sage

## Abstract

Nine new microsatellite markers (SSR) were isolated from *Salvia officinalis* L. A total of 125 alleles, with 8 to 21 alleles per locus, were detected in a natural population from the east Adriatic coast. The observed heterozygosity, expected heterozygosity, and polymorphic information content ranged from 0.46 to 0.83, 0.73 to 0.93 and 0.70 to 0.92, respectively. New microsatellite markers, as well as previously published markers, were tested for cross-amplification in *Salvia brachyodon* Vandas, a narrow endemic species known to be present in only two localities on the Balkan Peninsula. Out of 30 microsatellite markers tested on the natural *S. brachyodon* population, 15 were successfully amplified. To obtain evidence of recent bottleneck events in the populations of both species, observed genetic diversity (*H*_E_) was compared to the expected genetic diversity at mutation-drift equilibrium (*H*_EQ_) and calculated from the observed number of alleles using a two-phased mutation model (TPM). Recent bottleneck events were detected only in the *S. brachyodon* population. This result suggests the need to reconsider the current threat category of this endemic species.

## 1. Introduction

The genus *Salvia* is one of the largest plant genera, with approximately 1000 species [1]. Of the approximately 250 species that are common in the Mediterranean region, 11 species belong to the *Salvia officinalis* group [2].

Dalmatian, or common, sage (*S. officinalis* L.) is economically one of the most prominent species of the *Salvia officinalis* group [3]. It is an outcrossed, insect-pollinated, long-lived and sub-shrubby plant species that is used as an herb with beneficial healing properties [4], as an aromatic plant in the meat industry and for the treatment of inflammation [5]. Its economic importance has grown rapidly over the last several years. Moreover, it has recently begun to be used as an ornamental garden plant [6]; therefore, several cultivars have been developed for that purpose. Due to its many uses, Dalmatian sage is common in the Mediterranean region and all over the world. Although knowledge and use of Dalmatian sage dates to the Greek era, there is remarkable confusion concerning its taxonomy, distribution and variability in its natural range [7].

**Figure 1:**
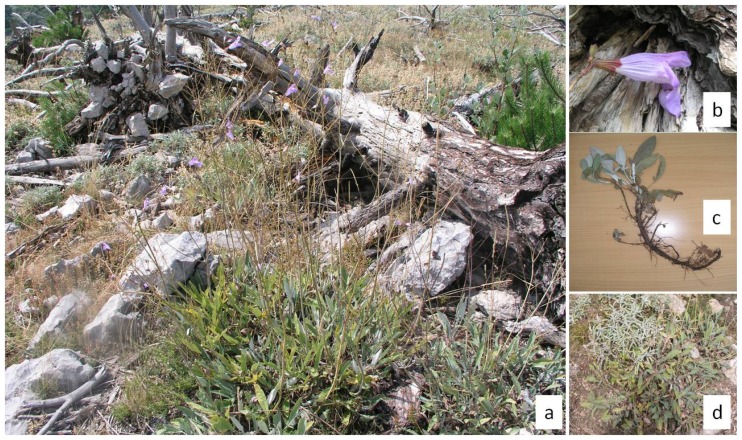
Ecological and morphological characteristics of short-tooth sage (*Salvia brachyodon* Vandas) on the Pelješac peninsula. (**a**) Dense patches of short tooth sage individuals spread quickly after a black pine forest fire in 1998; (**b**) Flowers up to 4 cm long; (**c**) Stolons below ground indicate possible clonal propagation; (**d**) Two congeners grow side by side with different flowering times (left upper corner = common sage, right lower corner = short tooth sage).

**Figure 2:**
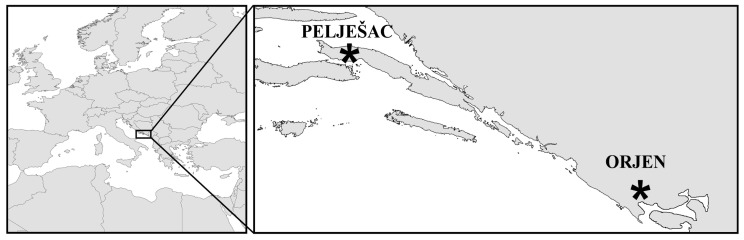
Geographic locations of the only known living populations of short-tooth sage (*Salvia brachyodon* Vandas).

Short tooth sage (*S. brachyodon* Vandas) is one of the most interesting species of the *Salvia officinalis* group from ecological, biogeographical, conservation and phylogenetic points of view (Figure 1). This is a relict species with a very narrow distribution (Figure 2). Although older literature [8–10] indicates its presence in more localities, only two have been confirmed at the present time: Mt. Sveti Ilija on the peninsula of Pelješac (Republic of Croatia) and 150 km southeast of Mt. Orijen (Republic of Bosnia and Herzegovina and Republic of Montenegro) [11–13]. Like many other members of this group, short-tooth sage is also rich in essential oils [14,15], and it is locally recognized and collected, especially in the region of Mt. Orjen [11]. Because of its very limited distribution, habitat fragmentation, succession and potential environmental threats, especially fire and overexploitation, short-tooth sage is a highly vulnerable species. It is noteworthy that the Croatian Red Book [16] classifies this species as near threatened (NT), while in the Republic of Montenegro, it is considered endangered (EN) [17]. In both countries, short-tooth sage is protected by law.

The genetic structure of plant populations reflects the interactions of different processes, including shifts in distribution, habitat fragmentation, population isolation, mutations, genetic drift, gene flow, and selection [18,19]. Prior information about the genetic structure of natural plant populations is an irreplaceable starting point for successful conservation and sustainable gathering of wild resources as well as breeding programs and agricultural production. In recent decades, a number of DNA-based molecular marker systems have been developed for the investigation of genetic diversity. Microsatellite markers (simple sequence repeats, SSRs) are one of the most valued genetic markers because of their high variability, codominance and repeatability.

The primary goal of this research was to identify new microsatellite markers for Dalmatian sage and to establish, together with previously developed microsatellite markers, a set of SSR markers for future population genetics studies of this species. The secondary goal was to examine the possibility of using microsatellite markers developed for Dalmatian sage in population genetics studies of short-tooth sage. This second objective is highly dependent on the total number of available markers. To meet both goals and to demonstrate that the loci contain sufficient variation for individual discrimination, natural populations of each species from the Pelješac peninsula were studied.

## 2. Results and Discussion

### 2.1. Development of New Microsatellite Markers for Dalmatian Sage

In total, 3840 colonies were screened for dinucleotide repeats in Dalmatian sage (*Salvia officinalis* L.). After removing low quality reads, 235 unique sequences remained in a total length of 107,875 bp. Out of 235 unique sequences, 224 contained GA or GT microsatellite repeats. High quality PCR primer pairs were designed for 15 dinucleotide microsatellite loci. Eleven of these were polymorphic, while four were either monomorphic or did not amplify at all. Finally, nine primer pairs had suitable amplification patterns and signal intensity and were used to screen 25 individuals representing a natural Dalmatian sage population (Table 1). The screen resulted in a total of 125 alleles, 8 to 21 alleles per locus, with an observed heterozygosity from 0.46 to 0.83, and an expected heterozygosity from 0.73 to 0.93. The DNA sequences of these microsatellite loci were deposited into GenBank under accession numbers JX440363 to JX440371.

Eight out of nine microsatellites showed a high polymorphic information content (PIC) of more than 0.75. It should be noted that the PIC value of the remaining locus (SoUZ021) was rather high (0.70), indicating that all nine loci could be very useful in assessing the genetic diversity and population structure of Dalmatian sage. Three out of nine newly developed microsatellite markers (SoUZ022, SoUZ025 and SoUZ027) showed significant deviations from Hardy-Weinberg expectations (HWE) after application of the sequential Bonferroni corrections. These three loci also exhibited an overall excess of homozygotes and null allele frequencies using Brookfield’s formula [20]. They varied from 0.14 (SoUZ025) to 0.21 (SoUZ027). However, bearing in mind the distribution range of the tested *S. officinalis* population as well as the range of altitudes of sampled individuals, this heterozygote deficiency is more likely due to population structure than to locus-specific phenomenon (e.g., scoring error or null alleles). In accordance with this opinion, we recommend a more detailed sampling in future population genetic studies of Dalmatian sage than was performed in this study. If using this sampling design results in the same loci continuing to show an overall excess of homozygotes and null allele frequency, corrections for null alleles or their exclusion from the study can be used as a last resort [21–23].

### 2.2. Cross-Amplification in Narrow Endemic *Salvia brachyodon*

Including previously published di- and tri-nucleotide microsatellite loci [24,25] and the primers published in this study, we were able to test 29 Dalmatian sage microsatellite markers for cross-amplification in short-tooth sage. The amplification rate was 52%. The 15 successfully amplified microsatellite markers were used for in-depth analysis in natural populations of both species (Table 2). The development of new microsatellite markers, which were described earlier in this paper, proved to be entirely justified because as many as four of these markers were among the 15 polymorphic markers that were successfully amplified in short-tooth sage. A total of 87 alleles were observed across 15 loci. The number of alleles per locus ranged from 3 to 9, the observed heterozygosity ranged from 0.33 to 0.92, and the expected heterozygosity ranged from 0.33 to 0.84. Only two microsatellite loci (SoUZ026 and SoUZ002) had low polymorphic information contents (PIC) of 0.31 and 0.47. One of the 15 microsatellite loci exhibited significant deviations from HWE (SoUZ020) and the presence of null alleles with a frequency of 0.18. Five out of the 105 tests for linkage disequilibrium were significant (*p* < 0.01) after applying sequential Bonferroni corrections (SoUZ006/SoUZ007, SoUZ009/SoUZ011; SoUZ014/SoUZ009; SoUZ006/SoUZ009 and SoUZ014/SoUZ020). Deviations from HWE and linkage disequilibrium are the result of primer-site mismatch (null alleles), which are common in cross-amplified species, or as a consequence of specific population structures (e.g., clonality). It is noteworthy that during fieldwork, dense patches of S. brachyodon individuals and stolons at the soil surface or below ground were observed (Figure 1c). Therefore, it is very likely that clonal individuals exist in S. brachyodon populations and that a significant result for linkage disequilibrium is a consequence. However, in our sample set of 25 individual short-tooth sage plants, none were genetically identical. More intensive population genetics research and tests for clonal propagation [26,27] will allow us to elaborate on short-tooth sage reproduction and offer a better explanation for deviations from HWE and linkage disequilibrium observed in this study.

### 2.3. Population Genetics Parameters and Structures of Natural Populations of Two Closely Related Species

Based on our descriptive statistics (Table 2), we can conclude that the number of alleles as well as the expected heterozygosity or genetic diversity were prominently higher in Dalmatian than in short-tooth sage. Only 29 of 234 detected alleles were common to both species studied (Table 2). Because microsatellites are known to have very rapid evolutionary rates, prominent differences between Dalmatian and short-tooth sage in the length of alleles at each microsatellite locus were expected. Therefore, the alleles that are common to both species may be the same due to coincidence (identity-in-state) rather than because of a common origin (identity-by-descent).

This study is among those that have shown that rare species have less genetic variation than widespread species [28,29]. However, by virtue of selecting the most polymorphic microsatellites, number of alleles tends to be higher in the species from which they were originally developed [30–32] and it is impossible to estimate to which extent cross-amplification procedure itself also contributed to the reduced genetic variation observed in the population of short-tooth sage. If the occurrence of null alleles is uncommon, the microsatellites successfully amplified in a related species proved to be a very useful tool in population genetic studies. The greatest benefit of this study was the opportunity to explore population genetics phenomenon such as genetic bottleneck in rare and widespread congeners [33,34]. In this case, the differences in microsatellite variability between species do not influence the outcome of the analysis. The observed genetic diversity (*H*_E_) is compared to the expected genetic diversity at mutation-drift equilibrium (*H*_EQ_) in the same way regardless of the actual number of alleles at a locus and there is no advantage in using highly variable markers, as shown by the power analysis [35].

Tests for evidence of a recent bottleneck based on microsatellite loci and assuming the two-phased mutation model (TPM) were applied to both Dalmatian and short-tooth sage populations. A statistically significant departure from mutation-drift equilibrium was detected in short-tooth sage (Wilcoxon test; *p* = 0.02), suggesting that this population underwent a recent bottleneck that reduced its genetic diversity. The results of the same test, when applied to Dalmatian sage and based on 29 (all loci) or 15 (only those loci that were amplified in short-tooth sage) microsatellite loci, were not significant in either case (*p* = 0.99 and 0.81, respectively). This result was expected for Dalmatian sage population because this is a large population with an extended distribution range.

According to recent data, short-tooth sage from the Pelješac peninsula is considered a near threatened (NT) species of Croatian flora [16]. According to the IUCN Standards and Petitions Subcommittee [36], near threatened species are close to being qualified as vulnerable. Evidence of a genetic bottleneck in the population on the Pelješac peninsula should prompt new testing of IUCN quantitative criteria used in determining whether a species is truly endangered. If these criteria prove that short-tooth sage is truly threatened, then we hope that this study will contribute to better protection.

## 3. Experimental Section

This study was carried out on 25 Dalmatian sage and 25 short-tooth sage plants from the natural populations originating on the Pelješac peninsula (Croatia). New microsatellites for *S. officinalis* L. were developed from genomic DNA libraries enriched for GA and GT repeats as described earlier [37], but with several modifications.

Genomic DNA samples were extracted from dried leaves using the GenElute Plant Genomic DNA Miniprep Kit (Sigma-Aldrich, St. Louis, MO, USA). Nine restriction enzymes were used for genomic DNA digestion (*Hae*III, *Mse*I, *Sau*3AI, *Rsa*I, *Alu*I, *Hin*fI, *Eco*RV, *Bgl*II and *Eco*RI) (New Englands Biolabs, Ipswich, MA, USA). Single-stranded overhangs of restriction fragments were removed using mung bean nuclease and dephosphorylated using calf intestinal alkaline phosphatase (CIP) (New England Biolabs, Ipswich, MA, USA). Phosphorylated linkers were prepared from SNXfor and SNXrev primers using T4 polynucleotide kinase (Thermo Fisher Scientific Inc., Waltham, MA, USA) [38]. Ligation of linkers to DNA fragments was performed by combining double-stranded SNX linkers, DNA fragments, *Xmn*I restriction enzyme and T4 DNA ligase (New England Biolabs, Ipswich, MA, USA). Long (GA)*_n_* and (GT)*_n_* probes were constructed in a PCR extension reaction, followed by their attachment to small (5 × 5 mm) pieces of nylon membrane (Nytran^®^ Super Charge, Schleicher & Schuell BioScience GmbH, Dassel, Germany) and overnight hybridization of DNA fragments containing microsatellite regions. After the nylon membranes were rinsed, the microsatellite fragments were ligated into the pGEM-T Easy Vector (Promega Corporation, Madison, WI, USA), and heat-shock transformation into XL10-Gold Ultracomponent Cells (Agilent Technologies, Stratagene, La Jolla, CA, USA) was performed. The resulting culture was spread on LB-agar plates containing ampicillin, IPTG and X-gal. After overnight incubation, white bacterial colonies were transferred by toothpick into 384-well plates containing Luria-Bertani (LB) freezing media (LB broth + 13 mM KH_2_PO_4_, 36 mM K_2_HPO_4_, 1.7 mM sodium citrate, 6.8 mM (NH_4_)_2_SO_4_, 4.4% *v*/*v* glycerol) for long-term storage. Libraries were transferred onto nylon membranes and screened by Southern analysis using Cy5- and Cy3-labeled 30 bp oligonucleotides with microsatellite core repeats. Positives were detected after stringent washing by scanning the blots using an Ettan DIGE Imager (GE Healthcare Biosciences, Pittsburgh, PA, USA).

Positive clones were randomly selected from the libraries and used for plasmid isolation (Wizard Plus SV Minipreps, Promega Corporation, Madison, WI, USA). Sequencing of plasmid isolates was performed by means of T7 and SP6 universal primers using Big Dye chemistry and an ABI 3730XL analyzer (Applied Biosystems, Foster City, CA, USA). Sequences were edited and assembled using CodonCode Aligner software version 2.0.6 (CodonCode Corporation, Dedham, MA, USA). Microsatellite repeats in sequences were localized by MISA PERL SCRIPT [39]. PCR primer pairs flanking microsatellite repeats were designed using the PRIMER 3 program [40]. Because some of the SSR markers could have been monomorphic or might not have amplified well, new microsatellite PCR primers were first tested on five randomly chosen Dalmatian sage DNAs. Only polymorphic SSR markers with good amplification were tested on the complete set of DNA samples from both natural populations studied using a tailed primer protocol [41]. PCR amplification was performed on the GenAmp^®^ PCR System 9700 (Applied Biosystems, Foster City, CA, USA) using a two-step protocol with an initial touchdown cycle. The cycling condition were as follows: 94 °C for 5 min; five cycles of 45 s at 94 °C, 30 s at 60 °C for the first cycle and 1 °C less in each subsequent cycle, and 90 s at 72 °C; 25 cycles of 45 s at 94 °C, 30 s at 55 °C, and 90 s at 72 °C; and an 8 min extension step at 72 °C. Three different lebeled PCR products (6-FAM, VIC, NED) and 500-LIZ size standard were collected and run as a single sample on an ABI 3730XL (Applied Biosystems, Foster City, CA, USA). The results were analyzed using GeneMapper 4.0 software (Applied Biosystems, Foster City, CA, USA).

For each microsatellite locus, the average number of alleles per locus (*N*_a_), the observed heterozygosity (*H*_O_), the expected heterozygosity or genetic diversity (*H*_E_), and the polymorphism information content (PIC) [42] were calculated using PowerMarker V3.23 [43]. GENEPOP version 3.4 [44] was used to test genotypic frequencies for conformance to Hardy-Weinberg expectations (HWE) and to test the loci for gametic disequilibrium (LD). Sequential Bonferroni adjustments [45] were applied to correct for the effect of multiple tests using SAS release 8.02 (SAS Institute Inc., Cary, NC, USA). Each locus was evaluated for the presence of null alleles, scoring errors, and allelic dropout using Micro-Checker version 2.2.3 [46]. The program BOTTLENECK version 1.2.02 [35] was used to test for evidence of recent bottleneck events in the populations of both species on the basis of this theoretical expectation [47]. The observed genetic diversity (*H*_E_) was compared to the expected genetic diversity at mutation-drift equilibrium (*H*_EQ_) and calculated from the observed number of alleles under the intermediate Two-Phase Model (TPM), assuming 30% multistep changes. The Two-Phase Model was applied because it has been shown to be the most appropriate for microsatellite DNA data [48]. Based on the number of loci in our dataset, the Wilcoxon signed-rank test [49] was chosen for the statistical analysis of heterozygote excess or deficiency, as recommended by Piry *et al.* [47].

## 4. Conclusions

Nine new microsatellite markers (SSR) were isolated from Dalmatian sage (*Salvia officinalis* L.). The observed parameters indicate that all loci may be very useful for assessing genetic diversity and population structures of Dalmatian sage. New microsatellite markers, as well as previously published markers, were tested for cross-amplification in short-tooth sage (*Salvia brachyodon* Vandas). The amplification rate was 52%. The greatest benefit of this study was the opportunity to compare genetic variation in rare and widespread congeners. This study is among those that have shown that rare species have less genetic variation than widespread species. Recent bottleneck events detected in the short-tooth sage population using a two-phased mutation model (TPM) highlight the need to reconsider the current threat category of this endemic species.

## Figures and Tables

**Table 1:** Characteristics of nine new microsatellite markers and results of primer screening on 25 individuals from a natural population of *Salvia officinalis* L. from the Pelješac peninsula.

Locus name	Repeat motif	Primer sequence (5′–3′)	Size (bp)	*N*_a_	*H*_O_	*H*_E_	PIC	GenBank accession No.
SoUZ021	(CA)_13_	F: CATTCTTTGCAGGGATTCGT R: GATGCTTCCTCGGCTGACTA	226–242	8	0.50	0.73	0.70	JX440363
SoUZ022	(AG)_19_	F: TCTTCGAGCCTGGAGTTTT R: AGAAGCAAGACAACCCCAAA	226–264	18	0.54	0.89*	0.89	JX440364
SoUZ023	(AC)_14_	F: CCTGCAAAACACAAACGAA R: GTTGTTTCGCTGGTGATGAA	171–185	8	0.83	0.83	0.81	JX440365
SoUZ024	(GA)_25_	F: TGGTCGTGTTGAACTTTCG R: AAGGAAGGTGCACCAAAATG	128–177	18	0.65	0.89	0.89	JX440366
SoUZ025	(AG)_31_	F: AGGTGTGTGACCCTGCTATG R: GGTTTTGCTCCATTGCATTT	205–246	21	0.67	0.93*	0.92	JX440367
SoUZ026	(AG)_17_	F: TTCATCTTTGACCGGAAAAC R: CATGTGGTGATGCGAGATTC	160–191	13	0.67	0.86	0.85	JX440368
SoUZ027	(AG)_24_	F: GGCGAGATTCATTTCCTTGA R: CATCAGTGAGGCTTGGTTCA	196–240	14	0.46	0.84*	0.83	JX440369
SoUZ028	(AG)_19_	F: GGGCCTTGTCTGCATGTATT R: TCCGGCGATTGTTCTCTAAT	201–235	15	0.71	0.88	0.87	JX440370
SoUZ029	(GT)_13_	F: AAACACGCATTTGTACGTGAA R: CCAACGACAACATCATCGTC	155–182	10	0.52	0.84	0.81	JX440371

*N*a = number of alleles; *H*O = observed heterozygosity; *H*E = expected heterozygosity; PIC = polymorphic information content;

* significant deviations from Hardy-Weinberg equilibrium after sequential Bonferroni corrections at the 0.1% nominal level.

**Table 2:** Summary of the microsatellite amplifications in *Salvia officinalis* and *Salvia brachyodon* populations from the Pelješac peninsula.

Locus	*Salvia officinalis* (*N* = 25)					*Salvia brachyodon* (*N* = 25)					
	
	Size (bp)	*N*_a_	*H*_O_	*H*_E_	PIC	Size (bp)	*N*_a_	*H*_O_	*H*_E_	PIC	CA
SoUZ001^a^	159–195	15	0.83	0.90	0.90	158–172	4	0.72	0.66	0.62	-
SoUZ002^a^	199–222	11	0.60	0.77	0.74	190–194	3	0.67	0.54	0.47	-
SoUZ004^a^	195–227	12	0.62	0.82	0.80	199–212	6	0.58	0.60	0.57	2
SoUZ005^a^	131–160	9	0.30	0.81*	0.79	122–130	5	0.83	0.77	0.73	-
SoUZ006^a^	204–244	14	0.83	0.88	0.87	204–233	9	0.76	0.84	0.82	6
SoUZ007^a^	214–227	8	0.58	0.72	0.69	210–240	9	0.92	0.84	0.82	5
SoUZ008^a^	190–210	10	0.71	0.80	0.77	176–180	3	0.80	0.65	0.57	-
SoUZ009^a^	209–250	9	0.61	0.74	0.70	220–235	6	0.72	0.73	0.70	2
SoUZ011^a^	175–227	19	0.76	0.93	0.93	230–250	6	0.92	0.77	0.74	-
SoUZ014^b^	201–237	12	0.87	0.88	0.87	192–218	9	0.75	0.82	0.80	2
SoUZ020^b^	198–246	10	0.50	0.77	0.74	199–217	4	0.33	0.62*	0.55	-
SoUZ021	226–242	8	0.50	0.73	0.70	224–238	5	0.58	0.69	0.65	4
SoUZ023	171–185	8	0.83	0.83	0.81	167–183	6	0.75	0.76	0.72	2
SoUZ024	128–177	18	0.65	0.89	0.89	116–134	8	0.83	0.75	0.72	2
SoUZ026	160–191	13	0.67	0.86	0.85	166–176	4	0.37	0.33	0.31	4

*N* = number of individuals; *N*_a_ = number of alleles; *H*_O_ = observed heterozygosity; *H*_E_ = expected heterozygosity; PIC = polymorphic information content; CA = common alleles;

a = previously published di-nucleotide microsatellite loci [24];

b = previously published tri-nucleotide microsatellite loci [25];

* = significant deviations from Hardy-Weinberg equilibrium after sequential Bonferroni corrections at the 0.1% nominal level.
